# Women from rural Guatemala who speak Mayan languages have reduced odds of diabetes, hypertension and obesity

**DOI:** 10.3389/fpubh.2025.1616498

**Published:** 2025-07-09

**Authors:** Stephen Alajajian, Anahí Venzor Strader, Yolanda Juarez Martin, Caitlin Scott, Peter Rohloff

**Affiliations:** ^1^Centro de Investigación en la Salud Indígena, Wuqu' Kawoq, Tecpán, Guatemala; ^2^Boston Children's Hospital, Division of Emergency Medicine, Boston, MA, United States; ^3^The Friendship Bridge, Lakewood, CO, United States; ^4^Division of Global Health Equity, Brigham and Women's Hospital, Boston, MA, United States

**Keywords:** Guatemala, Indigenous, Mayan, languages, diabetes, hypertension, obesity, health

## Abstract

**Introduction:**

Indigenous languages are integral to the individual and collective identity of humankind. Health benefits of speaking Indigenous languages have been demonstrated but may also be masked by various forms of linguistic and ethnic discrimination. Guatemala has experienced a significant degree of Mayan language loss and endangerment in recent decades. Recognition of the positive associations between Mayan languages and health may positively influence their trajectory.

**Methods:**

We undertook a cross-sectional analysis of a pre-existing dataset from a clinical population of women from Central and Western Guatemala. We compared prevalence of diabetes, hypertension, obesity and underweight among Mayan- and Spanish-speaking Indigenous women, and among non-Indigenous women. We used multiple logistic regression to estimate adjusted odds ratios for each condition by language preference, controlling for confounding factors.

**Results:**

A total of 10,876 women were included in the analysis. Indigenous speakers of Mayan languages had the lowest prevalence of diabetes, hypertension and obesity, and non-Indigenous women had the highest prevalence of underweight. After controlling for sociodemographic factors, Mayan language preference was associated with decreased odds of diabetes [aOR 0.80, 95%CI (0.67, 0.94)], hypertension [aOR 0.80, 95%CI (0.71, 0.91)] and obesity [aOR 0.82, 95%CI (0.74, 0.90)].

**Discussion:**

The reduced odds of diabetes, hypertension and obesity among women who prefer to speak Mayan languages might be explained by cultural and lifestyle factors that are inextricably tied to Mayan language use. These findings are consistent with several previous studies, although associations between Indigenous languages and obesity have been varied. Our findings strengthen the impetus to maintain the vitality of Mayan languages in Guatemala.

## Introduction

Indigenous languages are essential to the collective and individual identity of humankind (1). More than a communication tool, they are avenues for ancestral knowledge and ways of doing (1, 2). Nevertheless, a staggering number of Indigenous languages are disappearing (1). The exclusion, marginalization and devaluing of Indigenous language and culture have played and continue to play a significant role in their decline (2, 3). Furthermore, pressures to abandon Indigenous language use and traditional ways of life in a globalized world are many. Greater recognition of the positive associations between Indigenous language use and individual and collective wellbeing could positively influence the trajectory of Indigenous languages throughout the world.

Multiple studies have demonstrated the health benefits of using Indigenous languages (4–7). Whalen et al. conducted a review that identified 130 publications describing health outcomes, using both Western and Indigenous definitions of health, for Indigenous people who did and those who did not learn or use their ancestral language. They found that ancestral language learning or use was associated with better health outcomes in a majority of the publications reviewed (4). This is not surprising given the role of Indigenous languages in strengthening a sense of collective identity and conveying medicinal, ecological, and other traditional knowledge (4).

The health benefits of speaking Indigenous languages could be countered by various forms of discrimination. Speakers of Indigenous languages are often unable to receive healthcare in their own language, and they are often treated poorly in healthcare settings if they do not speak the dominant language (8). Furthermore, public health research and global health promotion efforts may discount Indigenous languages or exclude speakers from participation (9). It is known that Indigenous people in general face worse health outcomes than non-Indigenous people for a variety of reasons that include poverty, political marginalization and ethnic discrimination (8). For example, a recent analysis of cross-sectional data on 186,393 children aged 3–4 years from 40 countries found Indigenous and minority language use to be associated with poorer childhood development scores compared to dominant language use (10). However, it can be difficult to disentangle the effects of language-related factors and ethnicity-related factors on health and wellbeing, as ethnic inequalities could mask the protective effects of Indigenous languages.

To our knowledge, no studies have specifically investigated the relationship between Mayan language use and health outcomes in Guatemala, a central American nation where nearly half the population identifies as Indigenous (11). Guatemala is home to 22 Mayan languages. Despite historical and ongoing efforts to erode the cultural identity of Mayan speakers, from the Spanish conquest to neocolonial influences and the atrocities of the civil war, these languages have demonstrated tremendous resilience (3). Nevertheless, in recent decades Guatemala has experienced a significant degree of Mayan language loss and endangerment (12). Among those who speak Mayan languages, older adults are the most likely to be monolingual speakers, and by extension, to have difficulty accessing healthcare in their own language (12). Health services in the public system are usually provided exclusively in Spanish (12, 13).

In this context, we examine the relationship between Mayan language preference and the prevalence of four health conditions in a clinical population of primarily rural and Indigenous women from 9 departments in Central and Western Guatemala. We compare the prevalence of diabetes, hypertension, obesity, and underweight across ethnicity and language groups and use multiple logistic regression to estimate the associations between Mayan language preference and the prevalence of each condition after controlling for ethnicity and other confounding factors.

## Methods

We conducted a cross-sectional analysis of a pre-existing dataset containing health and demographic data from a clinical population of women who participated in a non-governmental, community-based primary care program alongside a collaborating microfinance program in Central and Western Guatemala. Ethics approval was obtained from the Wuqu' Kawoq Institutional Review Board (WK-2022-004), and informed consent was waived after the protocol was determined to carry no more than minimal risk and to be infeasible without the waiver. Program details have been previously described (14). The initial dataset contained data extracted from the medical record between July 1, 2015 and December 31, 2022 and was limited to cases for which blood glucose data was available. For the present analysis, the study size was further decreased since we only included records for which language and ethnicity data were available. Participants were predominantly rural and Indigenous and came from the departments of Chimaltenango, Guatemala, Quetzaltenango, Quiché, Retalhuleu, Sacatepéquez, Sololá, Suchitepéquez, and Totonicapán.

The predictor variable of interest for this analysis was preferred language. The preferred languages reported by participants included Kaqchikel, K'iche', Mam, Sacapulteco, Tz'utujil, and Spanish, which were grouped into a binary variable (Spanish or Mayan language). The outcomes of interest were the prevalence of diabetes, hypertension, obesity and underweight. Diabetes was defined by self-reported history, use of glucose-lowering medication, or an elevated fasting (>= 126 gm/dl) or random (>= 200 mg/dl) glucose value. Hypertension was defined by self-reported history, use of blood pressure-lowering medication, or elevated systolic (>= 130) or diastolic (>= 80) pressure. Body mass index (BMI) was calculated by dividing weight in kilograms by height in meters squared. Obesity was defined as a BMI of 30 kg/m^2^ or more, and underweight was defined as a BMI of <18.5 kg/m^2^. Fasting and random glucose, systolic and diastolic blood pressure, and weight and height measurements were taken during primary care visits. Potential confounding variables included age at the time of data extraction, ethnicity (Indigenous or non-Indigenous) and poverty level. The latter was estimated using the Poverty Probability Index (PPI), based on reported asset ownership (15). Raw PPI scores were grouped into quartiles. Additional variables included setting (rural or urban) and income source, which we grouped into four categories (textiles, business, agriculture and other).

We used R version 4.4.1 and RStudio “Chocolate Cosmos” Release for Windows for all data analyses. Descriptive reporting of outcome variables and demographic characteristics based on preferred language, stratified by ethnicity, was carried out using the *gtsummary* and *gt* packages. Outcome variables were reported as percentages with confidence intervals. For demographic variables, medians and interquartile ranges were reported for quantitative variables, while percentages and confidence intervals were reported for qualitative variables. Multiple logistic regression analysis was undertaken to control for potential confounding variables of age, ethnicity, poverty level, setting and income source. Given high proportions of missing data for key variables, multiple imputation with chained equations was run with 100 iterations using the *mice* package in R prior to performing multiple logistic regression. The *logreg* method was used for imputation of dichotomous variables and the *polyreg* method for imputation of categorical variables. Missing values were imputed for all variables included in the final regression analysis.

## Results

**Figure 1:**
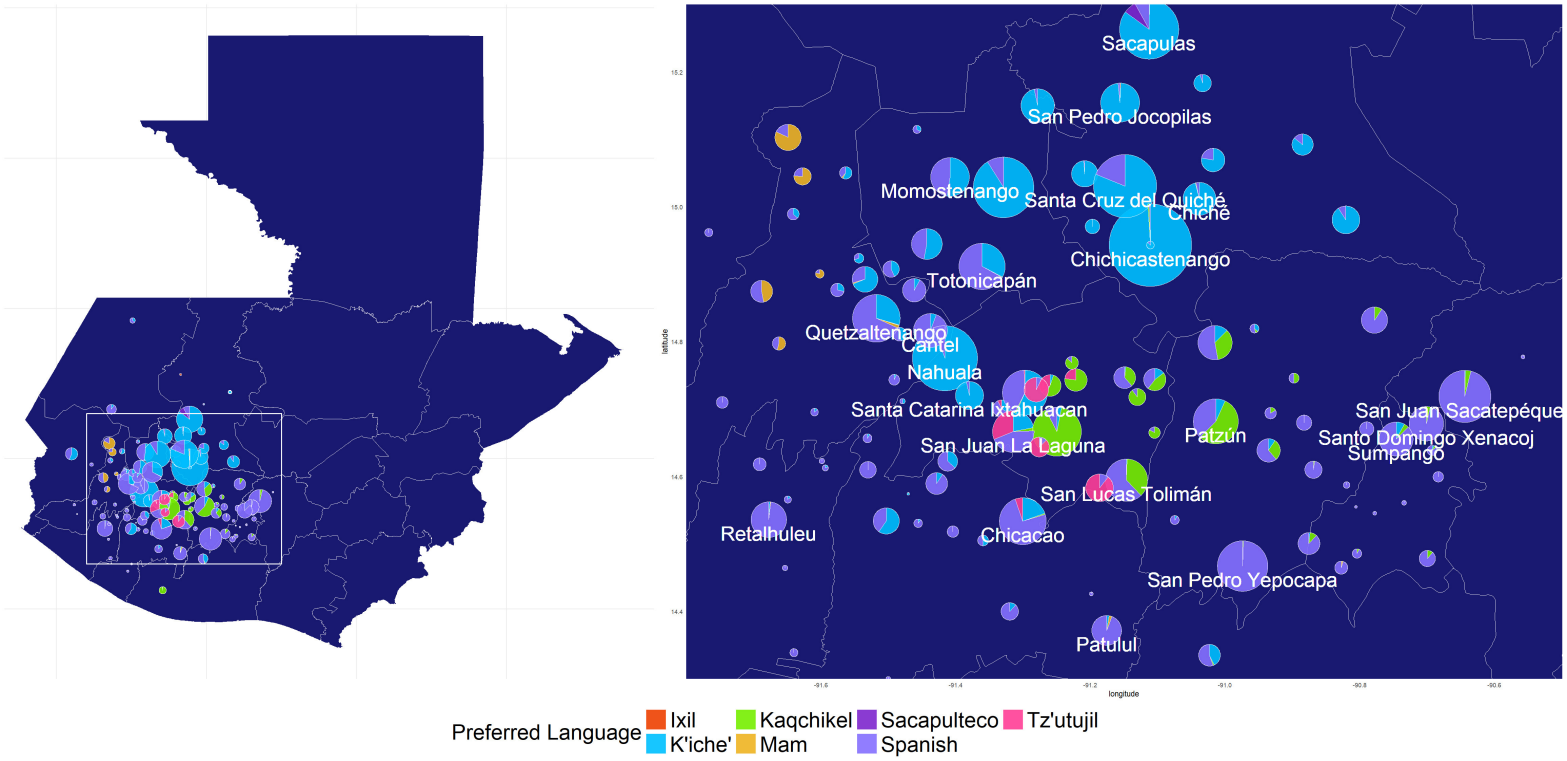
Geographical distribution of preferred language from a clinical population of primarily rural and Indigenous women in Western and Central Guatemala (*n* = 10,876). Pie chart size reflects the number of women recruited from each municipality.

The initial dataset contained 13,643 observations. After excluding observations for which data on language preference (2,765) and ethnicity (2) were not available, 10,876 observations remained. In total, 9,556 women identified as Indigenous, and 1,320 identified as non-Indigenous. Among those who identified as Indigenous, 6,414 preferred to speak a Mayan language, and 3,142 preferred to speak Spanish. Among the Indigenous women who preferred to speak a Mayan language, 4,872 spoke K'iche', 946 spoke Kaqchikel, 352 spoke Tz'utujil, 206 spoke Mam, 33 spoke Sacapulteco and 5 spoke Ixil. Among those who identified as non-Indigenous, all preferred to speak Spanish except for 10 who reported K'iche' (6) and Kaqchikel (4) language preference. Figure 1 shows the geographical distribution of the preferred languages of women in the dataset. Data were missing for the following variables: poverty level (3,705), source of income (3,527), setting (3,464), number of children (472), body mass index (143), obesity (143), underweight (143) and hypertension (7).

Demographic characteristics of non-Indigenous women, Indigenous speakers of Spanish, and Indigenous speakers of Mayan languages were similar for age and household size, but differed for poverty level, setting and source of income (Table 1). Indigenous women more commonly lived in rural settings than non-Indigenous women, although setting did not differ significantly between Spanish and Mayan language speakers who identified as Indigenous. Mayan language-speaking Indigenous women were the most likely to be poor, followed by Spanish-speaking Indigenous women, followed by non-Indigenous women. Mayan-speaking Indigenous women had the highest percentage of textile work, followed by Spanish-speaking Indigenous women, followed by non-Indigenous women. Income from businesses followed the opposite trend.

**Table 1:** Demographic characteristics of a clinical population of 10,876 women participating in a microfinance program in Guatemala, by ethnicity and preferred language.

	**Indigenous**					**Non-indigenous**		
		**Mayan language** ***N*** = **6,414**		**Spanish** ***N*** = **3,142**			**Mainly Spanish**^c^ ***N*** = **1,320**	
**Characteristic**	***N***	**Median/N** ^a^	**95% CI** ^b^	**Median/N**	**95% CI**	**N**	**Median/N**	**95% CI**
Age	9,556	41 (32, 51)		37 (29, 48)		1,320	37 (29, 49)	
Household size	9,219	4 (2, 6)		3 (2, 5)		1,185	3 (2, 4)	
Setting	6,441					971		
Rural		3,748 (86%)	85%, 87%	1,805 (87%)	85%, 88%		732 (75%)	73%, 78%
Urban		612 (14%)	13%, 15%	276 (13%)	12%, 15%		239 (25%)	22%, 27%
Poverty quantile	6,398					773		
1-Least likely poor		552 (12%)	11%, 13%	420 (22%)	20%, 24%		282 (36%)	33%, 40%
2		1,203 (27%)	26%, 28%	729 (38%)	35%, 40%		312 (40%)	37%, 44%
3		1,163 (26%)	25%, 27%	424 (22%)	20%, 24%		120 (16%)	13%, 18%
4-Most likely poor		1,536 (34%)	33%, 36%	371 (19%)	17%, 21%		59 (7.6%)	5.9%, 9.8%
Source of income	6,389					960		
Agriculture		1,096 (25%)	24%, 27%	490 (24%)	22%, 26%		275 (29%)	26%, 32%
Business		1,493 (34%)	33%, 36%	1,045 (51%)	49%, 53%		614 (64%)	61%, 67%
Textiles		1,686 (39%)	37%, 40%	460 (22%)	21%, 24%		35 (3.6%)	2.6%, 5.1%
Other		61 (1.4%)	1.1%, 1.8%	58 (2.8%)	2.2%, 3.7%		36 (3.8%)	2.7%, 5.2%

a Median (Interquartile Range)/N (%).

b CI, Confidence Interval.

c Ten individuals of non-Indigenous ethnicity reported Mayan language preference.

As shown in Table 2, Indigenous speakers of Mayan languages had the lowest prevalence of diabetes (7.8%), hypertension (15%) and obesity (27%), followed by Indigenous speakers of Spanish (8.8%, 17% and 32%, respectively), followed by non-Indigenous women (11%, 24% and 37%, respectively). Mayan-speaking Indigenous women had a higher prevalence of underweight than Spanish-speaking Indigenous women (1.1% vs. 0.8%), while non-Indigenous women had the highest prevalence of underweight (1.5%) among all the groups. After controlling for sociodemographic factors in multiple logistic regression, Mayan language preference was associated with decreased odds of diabetes [aOR 0.80, 95%CI (0.67, 0.94)], hypertension (aOR 0.80, 95%CI (0.71, 0.91)] and obesity [aOR 0.82, 95%CI (0.74, 0.90)] as shown in Table 3. Diabetes was classified based on self-report alone in 13 cases (1.2% of 1,125 women classified as having diabetes) and hypertension was classified on the basis of self-report alone in 100 cases (4.3% of 2,330 women classified as having hypertension). No statistically significant association was found between Mayan language use and underweight.

**Table 2:** Prevalence of diabetes, hypertension, overweight and underweight in a clinical population of 10,876 women participating in a microfinance program in Guatemala, by ethnicity and preferred language.

	**Indigenous**					**Non-Indigenous**	
		**Mayan language** ***N*** = **6,414**		**Spanish** ***N*** = **3,142**			**Mainly Spanish**^a^ ***N*** = **1,320**	
**Condition**	***N***	**Prevalence**	**95% CI** ^b^	**Prevalence**	**95% CI**	***N***	**Prevalence**	**95% CI**
Diabetes	9,556	501 (7.8%)	7.2%, 8.5%	278 (8.8%)	7.9%, 9.9%	1,320	149 (11%)	9.7%, 13%
Hypertension	9,551	968 (15%)	14%, 16%	522 (17%)	15%, 18%	1,318	319 (24%)	22%, 27%
Underweight	9,435	69 (1.1%)	0.86%, 1.4%	25 (0.8%)	0.53%, 1.2%	1,298	19 (1.5%)	0.91%, 2.3%
Obese	9,435	1,714 (27%)	26%, 28%	1,010 (32%)	31%, 34%	1,298	486 (37%)	35%, 40%

a Ten individuals of non-indigenous identity reported Mayan language preference.

b CI = Confidence Interval.

Lower N for hypertension, underweight and obesity reflect missing data: 7 missing observations for hypertension and 143 missing observations for body mass index (BMI).

**Table 3:** Odds ratios for diabetes, hypertension, overweight and underweight in a multiple logistic regression analysis of a clinical population of 10,876 women participating in a microfinance program in Guatemala.

	**Diabetes (*****n*** = **10,876)**			**Hypertension (*****n*** = **10,876)**			**Obesity (*****n*** = **10,876)**			**Underweight (*****n*** = **10,876)**		
**Characteristic**	**OR** ^a^	**95% CI** ^a^	***p*** **-value**	**OR** ^a^	**95% CI** ^a^	***p*** **-value**	**OR** ^a^	**95% CI** ^a^	***p*** **-value**	**OR** ^a^	**95% CI** ^a^	***p*** **-value**
Age	1.07	1.06, 1.07	**<0.001**	1.06	1.05, 1.06	**<0.001**	1.01	1.00, 1.01	**<0.001**	0.99	0.97, 1.00	0.2
**Poverty quantile**												
1-Least likely poor	—	—		—	—		—	—		—	—	
2	0.95	0.77, 1.18	0.7	0.81	0.69, 0.95	**0.009**	0.87	0.76, 0.99	**0.029**	2.19	1.08, 4.46	**0.030**
3	0.83	0.66, 1.05	0.13	0.78	0.66, 0.93	**0.004**	0.78	0.68, 0.89	**<0.001**	1.86	0.89, 3.85	0.10
4-Most likely poor	0.68	0.52, 0.89	**0.004**	0.60	0.50, 0.73	**<0.001**	0.59	0.51, 0.68	**<0.001**	1.97	0.95, 4.09	0.070
**Ethnicity**												
Non-indigenous	—	—		—	—		—	—		—	—	
Indigenous	0.82	0.65, 1.02	0.079	0.67	0.56, 0.79	**<0.001**	0.84	0.73, 0.97	**0.016**	0.51	0.28, 0.94	**0.032**
**Setting**												
Rural	—	—		—	—		—	—		—	—	
Urban	0.96	0.77, 1.19	0.7	1.30	1.11, 1.52	**<0.001**	0.87	0.76, 1.00	**0.050**	1.11	0.62, 1.98	0.7
**Source of income**												
Agriculture	—	—		—	—		—	—		—	—	
Business	1.49	1.21, 1.82	**<0.001**	1.02	0.88, 1.19	0.8	1.10	0.98, 1.24	0.11	0.79	0.47, 1.33	0.4
Other	1.56	0.91, 2.67	0.11	1.10	0.74, 1.65	0.6	1.13	0.82, 1.57	0.4	1.06	0.25, 4.45	>0.9
Textiles	1.23	0.98, 1.55	0.071	1.10	0.93, 1.31	0.3	1.09	0.96, 1.24	0.2	0.84	0.48, 1.47	0.5
**Preferred language**												
Spanish	—	—		—	—		—	—		—	—	
Mayan language	0.80	0.67, 0.94	**0.008**	0.80	0.71, 0.91	**<0.001**	0.82	0.74, 0.90	**<0.001**	1.30	0.81, 2.09	0.3

a OR, Odds Ratio; CI, Confidence Interval. Bold values indicate statistical significance (*p* < 0.05).

## Discussion

In a cross-sectional analysis of 10,876 women who participated in a community-based primary care program alongside a microfinance program in Guatemala, Indigenous women who spoke Mayan languages had the lowest prevalence of diabetes [7.8%, 95%CI (7.2–8.5%)], hypertension [15%, 95%CI (14.0–16.0%)], and obesity [27%, 95%CI (26.0–28.0%)] compared to Spanish-speaking Indigenous women and non-Indigenous women. After controlling for age, ethnicity, poverty level, setting and income source, the association between Mayan language preference and reduced odds of each of these conditions remained significant. Reductions in odds for each condition were modest, ranging from 18% reduced odds for obesity to 20% reduced odds for diabetes.

We point out some important limitations of our study. First, the patient population was composed of primarily rural and Indigenous women who participated in a microfinance program with specific recruitment criteria, limiting generalizability to the broader population. Second, causal conclusions cannot be definitively drawn from this cross-sectional analysis. Third, no data were available on monolingualism, which is relevant in our setting given that monolingual Mayan language speakers would be the least able to access healthcare in their own language. Fourth, the quality of the sociodemographic data obtained through medical records could not be verified, and the small percentage of outcomes data which is self-reported may be unreliable. Fifth, our analysis does not distinguish between the severity of disease. Finally, we recognize the inherent limitations of BMI as an isolated diagnostic marker of clinical obesity and that the standard BMI thresholds we used may not be the optimal thresholds for this specific population.

Our study also has significant strengths. First, to our knowledge, it is the first study to investigate the relationship between Mayan language use and health outcomes in Guatemala, to demonstrate reduced odds of chronic disease and obesity among Mayan language speakers, and to investigate the relationship between Indigenous language use and hypertension. Second, the large size of our analytical dataset, containing over 10,000 observations, allows for sufficient statistical power to detect relationships that may have otherwise been undetectable. Third, the use of multiple logistic regression to control for ethnicity, age and other confounding factors strengthens the confidence that the inverse associations between language preference and the outcomes of interest are not spurious.

Consistent with our findings, two other studies found associations between Indigenous language use and diabetes that were suggestive of protective effects. A quantitative analysis of First Nations health data found that, at the community level, Indigenous language knowledge was a significant negative predictor of diabetes prevalence after adjustment for socioeconomic factors (β = −0.973, *p* =0.007) (5). Also, Indigenous language use was found to be a protective factor against progression from pre-diabetes to diabetes in the Upper North Island of New Zealand (7).

Our finding that Mayan language preference was inversely associated with obesity is consistent with the findings of a cross-sectional multivariate analysis of 2,592 Inuit adults. In this analysis, overweight and obesity were found to be inversely correlated with the use of an Inuit language as the primary language spoken in the home (59% prevalence among monolingual Inuit speakers vs. 71% and 73% prevalence for bilingual and monolingual English speakers, p <0.001) (6). Conversely, fluency in an Inuit language was found to be positively associated in multivariate analysis with some indices of obesity in the central Canadian Arctic (16). Also, Métis girls aged 11–14 who spoke an Aboriginal language in Canada were found to have a higher odds ratio [2.18, 95%CI (1.66–2.84)] for obesity after controlling for other factors, although the same relationship was not found for other age ranges or for boys (17). These contrasting findings point to the complexity of contextual and intrinsic factors unique to each Indigenous population.

In the present study, a pattern emerged in which Mayan language-speaking Indigenous women had the lowest prevalence of diabetes, hypertension, and obesity, followed by Spanish-speaking Indigenous women, and subsequently by non-Indigenous women. A potential explanation we propose is that women who remained more closely tied to the language and its associated way of life tended to preserve diet- and lifestyle-related cultural practices that are beneficial to health. Additionally, protective effects may have stemmed from a shared sense of cultural identity, which would presumably have been strongest among women who spoke Mayan languages and who identified as Indigenous. Alternatively, the exclusive or preferential use of Mayan languages for communication may have had an insulating effect against negative influences of the predominant globalized culture, such as the infiltration of fast food and ultra-processed food into rural areas, or may be reflective of less assimilation into the predominant culture. Further qualitative research may help to uncover explanations for these findings. It is also important to note that the non-Indigenous women within this predominantly Indigenous population, while unlikely to be representative of the overall non-Indigenous population of women in Guatemala, constitute a subgroup of women who appear to be at significantly higher risk for diabetes, hypertension, obesity and underweight.

In conclusion, we found that Mayan language preference was associated with reduced odds of diabetes, hypertension, and obesity in a clinical population of 10,876 Guatemalan women and that the relationship was significant after controlling for ethnicity, age, poverty level, setting and income source in multiple logistic regression analysis. This positive association between Mayan language preference and health was observed despite known obstacles to accessing healthcare for non-Spanish speakers in Guatemala. These findings strengthen the impetus to maintain the vitality of Mayan languages in Guatemala and add to a growing body of research that investigates the relationship between Indigenous language use and health in different contexts.

## Data Availability

The datasets presented in this article are not readily available due to privacy concerns. Requests to access the datasets should be directed to stephen@wuqukawoq.org.
